# Mode-Splitting for Refractive Index Sensing in Fluorescent Whispering Gallery Mode Microspheres with Broken Symmetry

**DOI:** 10.3390/s18092987

**Published:** 2018-09-07

**Authors:** Yvonne Q. Kang, Alexandre François, Nicolas Riesen, Tanya M. Monro

**Affiliations:** School of Engineering, University of South Australia, Mawson Lakes, SA 5095, Australia; yvonne.kang@unisa.edu.au (Y.Q.K.); nicolas.riesen@unisa.edu.au (N.R.); tanya.monro@unisa.edu.au (T.M.M.)

**Keywords:** whispering gallery mode, optical resonators, optical sensing and sensors

## Abstract

Whispering gallery mode (WGM) resonators have become increasingly diverse in terms of both architecture and applications, especially as refractometric sensors, allowing for unprecedented levels of sensitivity. However, like every refractometric sensor, a single WGM resonator cannot distinguish temperature variations from changes in the refractive index of the surrounding environment. Here, we investigate how breaking the symmetry of an otherwise perfect fluorescent microsphere, by covering half of the resonator with a high-refractive-index (RI) glue, might enable discrimination of changes in temperature from variations in the surrounding refractive index. This novel approach takes advantage of the difference of optical pathway experienced by WGMs circulating in different equatorial planes of a single microsphere resonator, which induces mode-splitting. We investigated the influence of the surrounding RI of the microsphere on mode-splitting through an evaluation of the sphere’s WGM spectrum and quality factor (Q-factor). Our results reveal that the magnitude of the mode-splitting increases as the refractive index contrast between the high-refractive-index (RI) glue and the surrounding environment increases, and that when they are equal no mode-splitting can be seen. Investigating the refractive index sensitivity of the individual sub modes resulting from the mode-splitting unveils a new methodology for RI sensing, and enables discrimination between surrounding refractive index changes and temperature changes, although it comes at the cost of an overall reduced refractive index sensitivity.

## 1. Introduction

Whispering gallery mode (WGM) resonators are small, axially symmetric dielectric devices, ranging from a few microns to hundreds of microns in diameter, with the unique ability to trap light by total internal reflection. The optical wave circulating along the resonator’s inner surface generates optical resonances, whose spectral positions are strongly dependent on the refractive index contrast between the resonator and its surrounding environment as well as the resonator’s shape [1]. This distinctive feature of WGMs, combined with their extremely high quality factor (Q-factor), defined as the ratio between the resonance wavelength and its full width at half maximum (FWHM), in addition to the small mode volume possible, have enabled WGM resonators to become prime candidates for highly sensitive, label-free bio-chemical sensors [1,2,3,4,5,6,7]. WGM based sensing mechanisms, such as (1) resonance spectral position shifts [3,4,5,6,7], (2) linewidth broadening/Q-factor spoiling of the resonances (e.g., due to stress-induced geometry deformation [8,9] or dissipative interaction [10,11,12]), and (3) splitting of the two counter-propagating WGMs [13,14], have been extensively researched. While unprecedented sensing performance, such as single molecule or particle detection (e.g., proteins in the range of fg/mL), has been demonstrated [4,5,10,13,14], the sensors are still complex to operate, requiring a phase-matched tapered fiber/or prism to excite and collect the WGMs. Furthermore, while single molecule detection is a tremendous achievement, with applications in fundamental research, it significantly restricts the dynamic range of the concentration where the sensor can be operated, thereby limiting its interest for the vast majority of medical diagnostic applications where detection of specific biomarkers, such as proteins, in the larger pg/mL to ng/mL range is required.

Active microresonators that are doped with a gain medium, allowing for remote (or free space) excitation and collection of the WGMs, are much easier to operate, and provide practical solutions [15] for biosensing applications where the analyte concentrations are within this range. Unlike passive microresonators, where WGMs from only one WGM plane are exploited at a time through phased-matched evanescent coupling schemes, active resonators, such as fluorescent microspheres, allow WGMs supported in all the equatorial planes to be collected and exploited simultaneously. Although this WGM collection approach can compromise the measured Q-factor of the resonator compared to the evanescent coupling approach [16], it offers some unique benefits in terms of refractive index sensing that are yet to be exploited. In particular, mode-splitting of the originally degenerate WGMs from different equatorial planes of a fluorescent microsphere due to environmental changes has not been investigated.

In this work, we break the symmetry of the environment surrounding a dye-doped microsphere by partially embedding the sphere into a high-refractive-index medium (i.e., glue) to lift the degeneracy of the modes from different WGM planes. The split modes from multiple planes of the fluorescent microsphere are then indiscriminately collected. This results in linewidth broadening of the overall resonance envelope and hence a reduction in the effective Q-factor. Further, by analyzing individual resonances, two sub-peaks were extracted, representing WGMs from two different equatorial planes that experience the lowest and the highest average RI coverage outside the sphere, respectively. The two sub-peaks exhibit different rates of wavelength shift per unit RI change. This unique effect can be exploited as a self-referenced sensing platform that can compensate for environmental effects, such as temperature fluctuations. This new approach is technically different from all the sensing methods reported previously and opens the door to a new class of self-referenced active WGM resonators, harnessing the RI mismatch between the probing environment and its own reference coating (e.g., glue) for biosensing and medical diagnostic applications.

## 2. Materials and Methods

### 2.1. Preparation of the Active Microsphere

**Figure 1:**
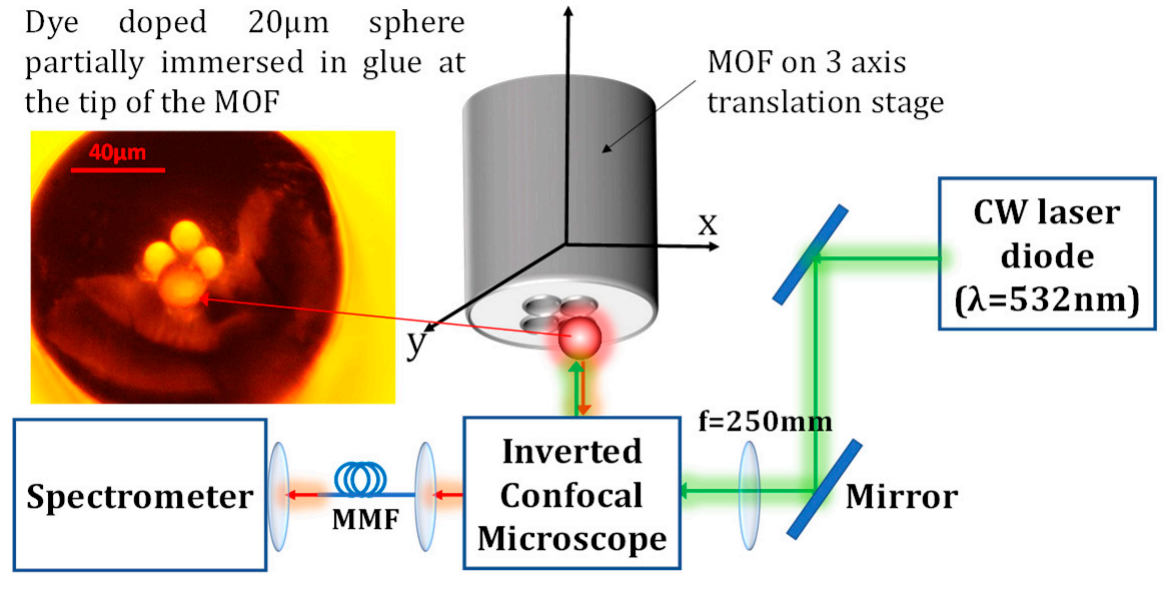
Schematic diagram of the experimental setup. MMF: Multi-mode fiber.

Polystyrene (PS) microspheres (*n*_ps_ = 1.59, Φ ~ 20 µm from Polysciences Inc., Warrington, PA, USA) were doped with Nile Red (*λ*_exc_ = 532 nm, *λ*_em_ ≈ 590 nm) using a two-phase liquid system described in our previous work [17]. The UV curing glue with a RI of ~1.382 at 590 nm once cured was purchased from Norland Products Inc., Cranbury, NJ, USA. A Microstructured-core Optical Fiber (MOF) contains four air holes of ~15 μm diameter towards the center (the details regarding the MOF design can be found in ref. [18]), and was used to hold the sphere and maneuver it into different liquids. We assembled the sensor in the following six steps. (a) A 10 cm freshly flat-cleaved MOF (held by a three-axis translational stage) was positioned above an inverted microscope. (b) The MOF was then carefully lowered down to lightly touch a droplet of fresh glue pipetted onto a glass coverslip, positioned on the inverted microscope (IX71, Olympus, Tokyo, Japan), allowing the glue to infiltrate the air holes through capillary forces. (c) A UV torch (*λ* = 365 nm, illumination: 300 mW/cm^2^) was used to partially cure the glue at the end of the MOF, thereby increasing its viscosity, and thus preventing it from flowing down and engulfing the whole sphere in the later stage. (d) A new glass coverslip with dried 20 μm microspheres resting on top was then placed on the inverted microscope. A microsphere was selected from many on the coverslip based on the criteria that the Q-factors of the fundamental WGMs must be above 2 × 10^4^ under the free space excitation and collection approach. (e) Once an appropriate microsphere was located, the MOF filled with partially cured glue was carefully lowered down to fetch the selected microsphere into one of its holes. (f) Lastly, the glue was fully cured using the UV torch to securely adhere the sphere to the hole of the MOF, as seen in the inset of Figure 1. The cured glue also provided a seal to prevent liquid from entering the holes of the MOF.

### 2.2. Optical Setup

A CW laser diode (*λ* = 532 nm, DJ532-40, Thorlabs Inc., Morganville, NJ, USA) was used for pumping the gain medium in the active microspheres. The free space laser beam was directed into the back port of the inverted microscope equipped with a 532 nm dichroic filter, effectively using the microscope as a confocal setup as shown in Figure 1. The WGM modulated fluorescence spectra from isolated microspheres deposited onto the microscope glass cover slip were observed through the microscope and spectrally resolved and recorded using a spectrometer (iHR550, Horiba, Kyoto, Japan) equipped with a cooled CCD (Synapse 2048 pixels, Horiba, Kyoto, Japan).

## 3. Results and Discussion

### 3.1. WGMs in Active Sphere Partially Covered by Glue

**Figure 2:**
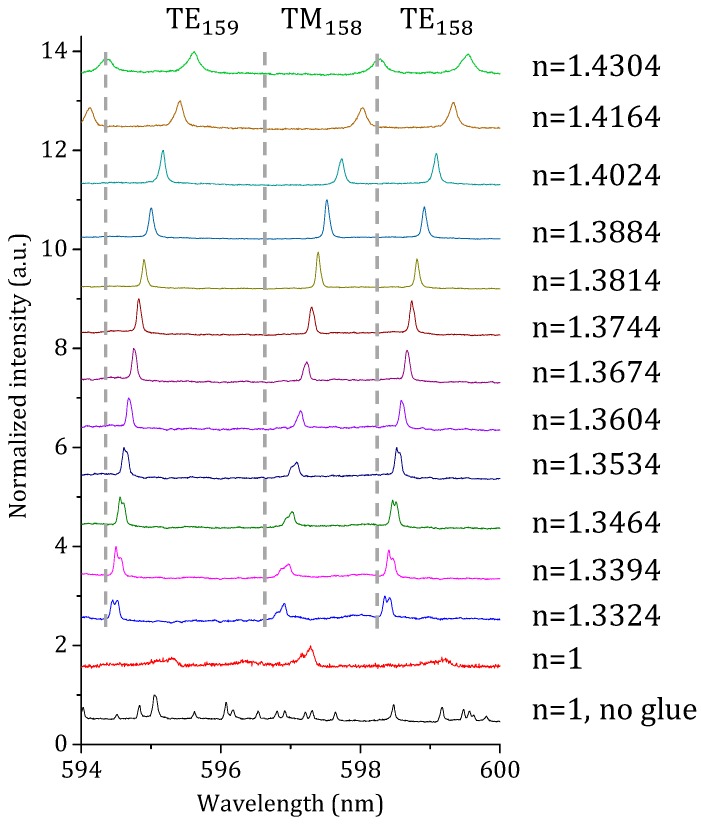
Normalized spectra of a single microsphere subjected to different environmental conditions. The different spectra were offset to highlight the resonance shift and Q-factor behavior. The black line indicates the fluorescence whispering gallery mode (WGM) spectrum of the sensing sphere before it was partially immersed in glue. The remaining spectra were taken in air and a range of liquids after the sphere was attached to the end of the Microstructured optical fiber (MOF) and partially covered by the glue.

The fluorescence WGM spectra of the single microsphere were collected before and after it was attached to the glue-filled MOF for comparison, as indicated by the black and red curves in Figure 2, respectively. Each spectrum in Figure 2 has been normalized and offset, providing a better overview of the WGM response as a function of the surrounding environment. Comparing the first two spectra (i.e., uncoated and coated microspheres, respectively), we can see that most of the resonance peaks disappeared, apart from the two prominent broad peaks in the wavelength region of 594 nm to 598 nm, when the free-standing sphere in air (black curve) became partially immersed in glue (red curve). The quenching and spectral behavior observed of the resonance peaks is due to three factors: (a) High-order modes are not supported in a ~20 μm PS microsphere if it is fully immersed in glue. Similarly, the high-order modes are expected to be mostly quenched in a partially-immersed sphere due to the increased average RI of the surrounding environment. (b) Given the large RI difference between the glue and air, both the first order TE and TM WGMs propagating along the inner surface of the sphere would experience greater scattering loss at the glue/air interfaces due to mismatched mode profiles. Consequently, the Q-factors of the fundamental TE and TM modes are severely compromised, and the TM mode would naturally suffer more due to its weaker confinement [19]. (c) The microsphere consists of an infinite number of equatorial planes where the WGMs can be supported. Owing to the partial coverage of the sphere by the glue, modes propagating along different planes exhibit slightly different effective optical path lengths. This essentially lifts the degeneracy between modes of different polar number, resulting in “mode-splitting,” in which the modes have different resonance wavelengths. In the case of active spheres, WGMs in all planes are excited simultaneously upon excitation with the pump laser, and the free space collection results in the indiscriminate sampling of a near-continuum of split-modes from all these equatorial planes. This results in linewidth broadening of the overall envelope of a given resonance and hence a reduction in the effective Q-factor. Similar “mode-splitting” effects have been reported in active microspheres with morphology imperfections (e.g., asphericity) [16]. With partial glue coverage, with the glue having an RI that is different to the probing environment, the strength of the “mode-splitting” is enhanced beyond that due to the inherent asphericity of the spheres.

Next, we collected 12 WGM spectra, also shown in Figure 2, as the MOF with sphere was dipped into liquids of RI ranging from *n* = 1.3324 (pure water) to 1.3884 (40% glycerol solution) at 0.0070 RI intervals, followed by ~0.0140 RI intervals to 1.4304 (70% glycerol solution). We conducted numerical simulations to fit the resonance positions (i.e., central wavelengths) of the fundamental TM and TE modes, shown in the three spectra of Figure 2, to gain an estimated value of the sphere size and the glue coverage. The numerical simulations solve the eigenvalue equations for the fundamental TM and TE modes in a microsphere and return the central wavelength positions of the modes of interest [19] based on the following inputs: Refractive index of the sphere, average refractive index (based on the weighted percentages of the glue and liquid) of the environment, diameter of the sphere, and wavelength range of interest. We selected three WGM spectra, where *n* = 1.3744, 1.3814, and 1.3884, which are those closest to the RI of the glue for all liquids tested. Using the measured WGM spectra in known glycerol solutions, the best fit can be found for the sphere size and the average coverage of the sphere after a standard iteration process. It was found that a sphere size of Φ ≈ 19.8 µm and an estimated glue coverage of 49% on the sphere surface provided the best fit of our experimental results. It is to be noted that the estimated glue coverage is for general guidance.

For *n* = 1.3814, we attributed the peak centered at ~597.52 nm to the fundamental TM_158_ mode, and the peak centered at ~598.92 nm to the fundamental TE_158_ mode. The grey dashed lines are used to divide the spectra for monitoring of the shifts of the TE_159_, TM_158_, and TE_158_ modes as the resonator is exposed to liquids from *n* = 1.3324 to *n* = 1.4304. One intriguing effect shown in Figure 2 is that the modes reappear as the sphere moves from air into liquid, which is contrary to what happens for uniform-surfaced microspheres (i.e., resonance quenching when a microresonator moves from air into liquid [20]). Reducing the RI difference between the liquid and the glue, by changing the surrounding liquid index from *n* = 1.3324 to *n* = 1.3814, results in clearly narrower modes as shown for the TM_158_ and TE_158_ modes in Figure 2. In particular, from *n* = 1.3324 to *n* = 1.3534, the TE_158_ mode can be seen to have two prominent sub-peaks that gradually merge into one. This is because as the RI difference between the probing liquid and the glue decreases, the strength of mode-splitting is reduced. As the RI of the liquid increases further, beyond that of the glue, the linewidths of the TM_158_ and TE_158_ modes begin to broaden again. We do not observe a doublet on either the TM_158_ or TE_158_ mode when *n* > 1.3814, observing only linewidth broadening. This is due to the high RI of the surrounding environment, which reduces the intrinsic Q-factor such that the strength of the mode-splitting is smaller than the broad linewidths of the individual sub-modes [20].

**Figure 3:**
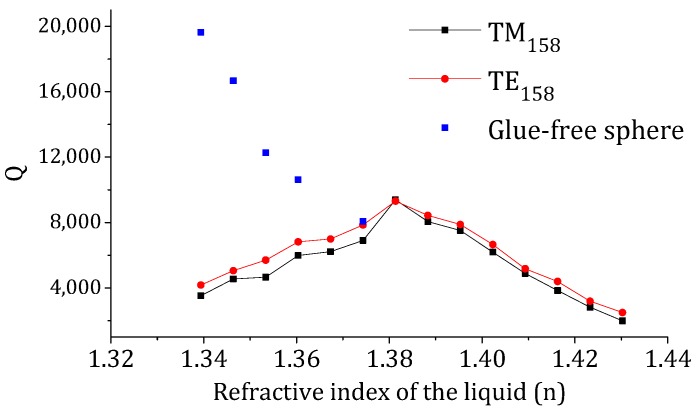
The effective Q-factors of the TM_158_ and TE_158_ modes as a function of the refractive index (RI) of the probing liquid. The blue dots represent the Q-factors as a function of the RI of the surrounding liquid for a free-standing 20 μm sphere taken from the same batch as the sphere partially covered in glue.

The effective Q-factors (*λ*/Δ*λ*) of the TM_158_ and TE_158_ modes as a function of the RI of the probing liquid are plotted in black and red dotted lines in Figure 3, respectively. For comparison, the Q-factor associated with the RI changes was independently characterized using a fresh glue-free microsphere from the same batch, and the results are shown in blue dots in Figure 3. As shown by the blue dots, for a glue-free uniform-surfaced microsphere, the WGM Q-factor decreases as the surrounding RI increases due to weaker light confinement inside the microsphere. In contrast, the effective Q-factors of both the TM_158_ and TE_158_ modes of the embedded microsphere increase as the RI of the liquid increases from *n* = 1.332 to *n* = 1.381. This implies RI non-uniformity induced mode-splitting has considerably stronger influence on the Q-factor than the change in the RI of the probing environment. At *n* = 1.381, where the RI of the probing liquid is closest to the RI of the glue, the Q-factors of both the TM_158_ and TE_158_ modes reach their highest values. As the RI of the probing environment increases beyond this turning point, the Q-factors of both the TM_158_ and TE_158_ modes start to decrease, at a rate that is expected to be faster than its uniform-surfaced counterpart, due to the additional mode-splitting contribution. We note that the RI difference of the probing environment relative to the reference glue could be calculated using the measured effective Q-factor of the resonator and the resonance spectral positions.

The strength of the mode-splitting can be evaluated from the RI difference, ∆*n_en_,* between the two WGM planes that see the highest and lowest effective RI of the local environment:

(1) $$\;\;n_{en\_high}=C_{high}n_{glue}+\left(1-C_{high}\right)n_{liquid}\;\;$$

(2) $$n_{en\_low}=C_{low}n_{glue}+\left(1-C_{low}\right)n_{liquid}\;$$

where *C_high_* is the glue coverage fraction on the WGM plane that sees the highest average RI, and *C_low_* is the glue coverage fraction on the WGM plane that sees the lowest average RI. Thus, ∆*n_en_* can be written as:

(3) $$\;\Delta n_{en}=\left(C_{high}-C_{low}\right)(n_{glue}-n_{liquid})\;$$

Equation (3) shows that both the distribution of the glue on the sphere surface and the RI difference between the reference glue and the surrounding liquid contribute to the strength of mode-splitting. The Q dependence on *n_liquid_*, i.e., the slope of the black or red dotted line in Figure 3, is due to this mode-splitting. From Equation (3), it can be inferred that before the fundamental TM or TE mode cutoff, the higher the RI of the glue and the larger the term *C_high_*–*C_low_*, the steeper the slope of the black (or the red) dotted line in Figure 3 would be.

**Figure 4:**
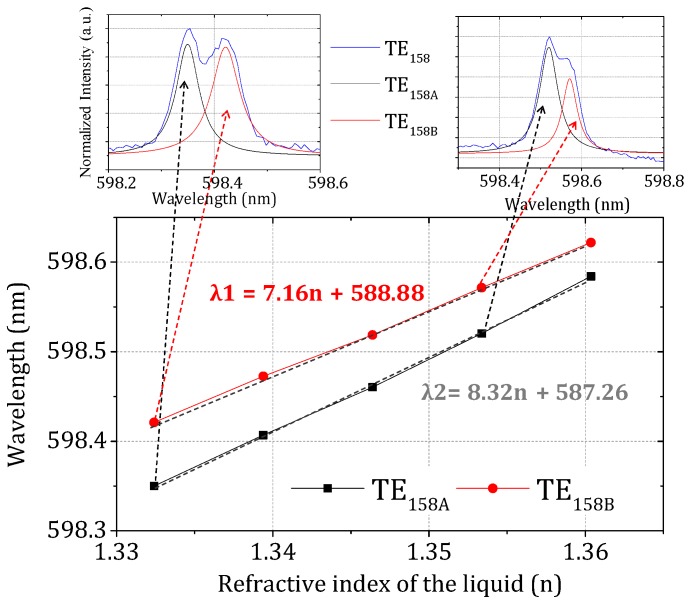
Measured resonance wavelengths as function of the surrounding RI for two sub-modes in TE_158_.

Finally, we took a close look at the shifts of the two prominent sub-peaks of the TE_158_ mode as the surrounding liquid RI changed from *n* = 1.332 to *n* = 1.353, which is within the dynamic range for most biomedical sensing applications, where the RI of the probing liquid is very close to that of water. By using multiple-peak Lorentz curve fitting of the global envelop of the TE_158_ mode, the central wavelengths of each sub-peak can be extracted and tracked, as shown in Figure 4. Here, we label the shorter and longer wavelength peaks as TE_158A_ and TE_158B_, respectively. By applying linear fits to the data in Figure 4, we found that the slopes of the two traces are slightly different, with the TE_158A_ mode having a larger slope, indicating a higher RI sensitivity. The physical origin of the difference between the two sub-modes comes from the different glue coverage percentages (relative to uncovered area) on their respective WGM planes. Clearly, the equatorial plane where the TE_158A_ mode circulates sees a lower glue coverage than the plane where the TE_158B_ mode lies in, and thus exhibits a higher sensitivity to RI changes in the aqueous environment. The fact that the two WGMs of the same mode order on the same resonator exhibit different sensitivities (due to different glue coverage) to the local environment opens the door to a self-referenced sensing platform.

### 3.2. A Self-Referenced Approach Based on Two Split-Modes

For a label-free biosensor, the ability to eliminate unwanted fluctuations in the signal, for example, due to changes in temperature, is crucial. For WGMs, the relationship between the wavelength shift and the changes in the RI of its surrounding environment can be simplified as Δ*λ*/*λ* = Δ*n_liquid_*/*n_liquid_* if the WGM resonator itself is assumed to have negligible dimensional nor RI change [21]. Environmental changes, such as temperature (i.e., Δ*T*), can contribute to Δ*n_liquid_*, and thus give a false reading. For the resonator presented in this work, the overall RI change of the surrounding environment for the two sub-modes, TE_158A_ and TE_158B_, assuming Δ*n_liquid_* and Δ*T*, is given by,

(4) $$\Delta n_{A}=\left(1-A\right)\cdot \Delta T\cdot T_{liquid}+\left(1-A\right)\cdot \Delta n_{liquid}+A\cdot \Delta T\cdot T_{glue}$$

(5) $$\Delta n_{B}=\left(1-B\right)\cdot \Delta T\cdot T_{liquid}+\left(1-B\right)\cdot \Delta n_{liquid}+B\cdot \Delta T\cdot T_{glue}$$

where *A* is the glue coverage fraction on the WGM plane A that supports the TE_158A_ mode, and *B* is the glue coverage fraction on the WGM plane B supporting the TE_158B_ mode. *T_liquid_* and *T_glue_* are the thermo-optic coefficients for the surrounding liquid (usually water) and the glue (or functional coating), respectively. Equations (4) and (5) can only be co-related if the modes, *A* and *B*, are of the same radial and azimuthal order on the same resonator, which is the case here for the split modes, TE_158A_ and TE_158B_. So under such conditions, the relative RI change between the modes, TE_158A_ and TE_158B_ (i.e., the strength of the mode-splitting), would be independent from the temperature impact on the resonator itself. The wavelength shifts, Δ*λ_A_* and Δ*λ_B_*, in relation to both Δ*n_liquid_* and the temperature change, Δ*T*, for the two sub-modes, TE_158A_ and TE_158B_, are then:

(6) $$\frac{\left(1-A\right)\cdot \Delta T\cdot T_{liquid}+\left(1-A\right)\cdot \Delta n_{liquid}+A\cdot \Delta T\cdot T_{glue}}{A\cdot n_{glue}+\left(1-A\right)\cdot n_{liquid}}=\frac{\Delta \lambda_{A}}{\lambda_{A}}$$

(7) $$\frac{\left(1-B\right)\cdot \Delta T\cdot T_{liquid}+\left(1-B\right)\cdot \Delta n_{liquid}+B\cdot \Delta T\cdot T_{glue}}{B\cdot n_{glue}+\left(1-B\right)\cdot n_{liquid}}=\frac{\Delta \lambda_{B}}{\lambda_{B}}$$

Therefore, in theory, if the glue (or functional coating) coverage percentages on the respective planes as well as the thermo-optic coefficients are known, Equations (6) and (7) become a system of two linear equations that when solved yield two variables, ∆*T* and ∆*n_liquid_*. Such a sensing platform will be able to discriminate temperature (i.e., Δ*T*) induced RI changes from an analyte concentration change (i.e., ∆*n_liquid_*) in the environment.

## 4. Conclusions

To summarize, we have investigated a novel WGM resonator platform, where part of the resonator is embedded into a high-refractive-index medium while the rest is left exposed to the surrounding environment. The intriguing behavior of the resulting WGM spectra reveals that the RI mismatch between the high RI medium and the surrounding environment can be evaluated from the effective Q-factor. It also opens the possibility for de-correlating temperature changes in RI sensing by analyzing the shifts of the subsets of the split modes. However, it comes at a significant cost in terms of refractive index sensitivity. We have previously shown that uncoated fluorescent and lasing microspheres can be used to routinely detect specific biomolecules down to few ng/mL concentrations, even in undiluted human serum [6,18]. Therefore, despite the significant reduction of both the refractive index sensitivity and Q-factor, we believe that such a platform would still be useful for a large range of biosensing applications where the target concentration ranges from hundreds of pg/mL to ng/mL.

The precise control of the distribution of the high RI coating (i.e., glue) on the surface of the microsphere is rather challenging using our current fabrication approach, although it is repeatable. However, we anticipate that 3D printing technologies could allow for precise deposition of the coating onto the microsphere to meet higher precision and reproducibility requirements for manufacturing.
